# Cavity-controlled methanol conversion over zeolite catalysts

**DOI:** 10.1093/nsr/nwad120

**Published:** 2023-05-05

**Authors:** Wenna Zhang, Shanfan Lin, Yingxu Wei, Peng Tian, Mao Ye, Zhongmin Liu

**Affiliations:** National Engineering Research Center of Lower-Carbon Catalysis Technology, Dalian National Laboratory for Clean Energy, iChEM (Collaborative Innovation Center of Chemistry for Energy Materials), Dalian Institute of Chemical Physics, Chinese Academy of Sciences, Dalian 116023, China; National Engineering Research Center of Lower-Carbon Catalysis Technology, Dalian National Laboratory for Clean Energy, iChEM (Collaborative Innovation Center of Chemistry for Energy Materials), Dalian Institute of Chemical Physics, Chinese Academy of Sciences, Dalian 116023, China; Energy College, University of Chinese Academy of Sciences, Beijing 100049, China; National Engineering Research Center of Lower-Carbon Catalysis Technology, Dalian National Laboratory for Clean Energy, iChEM (Collaborative Innovation Center of Chemistry for Energy Materials), Dalian Institute of Chemical Physics, Chinese Academy of Sciences, Dalian 116023, China; National Engineering Research Center of Lower-Carbon Catalysis Technology, Dalian National Laboratory for Clean Energy, iChEM (Collaborative Innovation Center of Chemistry for Energy Materials), Dalian Institute of Chemical Physics, Chinese Academy of Sciences, Dalian 116023, China; National Engineering Research Center of Lower-Carbon Catalysis Technology, Dalian National Laboratory for Clean Energy, iChEM (Collaborative Innovation Center of Chemistry for Energy Materials), Dalian Institute of Chemical Physics, Chinese Academy of Sciences, Dalian 116023, China; National Engineering Research Center of Lower-Carbon Catalysis Technology, Dalian National Laboratory for Clean Energy, iChEM (Collaborative Innovation Center of Chemistry for Energy Materials), Dalian Institute of Chemical Physics, Chinese Academy of Sciences, Dalian 116023, China; State Key Laboratory of Catalysis, Dalian Institute of Chemical Physics, Chinese Academy of Sciences, Dalian 116023, China; Energy College, University of Chinese Academy of Sciences, Beijing 100049, China

**Keywords:** methanol-to-olefins, cavity-controlled principle, reaction intermediates and routes, deactivation and diffusion, shape-selective catalysis

## Abstract

The successful development and application in industry of methanol-to-olefins (MTO) process brought about an innovative and efficient route for olefin production via non-petrochemical resources and also attracted attention of C1 chemistry and zeolite catalysis. Molecular sieve catalysts with diversified microenvironments embedding unique channel/cavity structure and acid properties, exhibit demonstrable features and advantages in the shape-selective catalysis of MTO. Especially, shape-selective catalysis over 8-MR and cavity-type zeolites with acidic supercage environment and narrow pore opening manifested special host–guest interaction between the zeolite catalyst and guest reactants, intermediates and products. This caused great differences in product distribution, catalyst deactivation and molecular diffusion, revealing the cavity-controlled methanol conversion over 8-MR and cavity-type zeolite catalyst. Furthermore, the dynamic and complicated cross-talk behaviors of catalyst material (coke)-reaction-diffusion over these types of zeolites determines the catalytic performance of the methanol conversion. In this review, we shed light on the cavity-controlled principle in the MTO reaction including cavity-controlled active intermediates formation, cavity-controlled reaction routes with the involvement of these intermediates in the complex reaction network, cavity-controlled catalyst deactivation and cavity-controlled diffusion. All these were exhibited by the MTO reaction performances and product selectivity over 8-MR and cavity-type zeolite catalysts. Advanced strategies inspired by the cavity-controlled principle were developed, providing great promise for the optimization and precise control of MTO process.

## Teaser

This review presents the most recent developments in the cavity-controlled reaction behavior of the methanol conversion catalyzed on the representative 8-MR and cavity-type zeolite.

## INTRODUCTION

The catalytic properties originating from the catalyst's microenvironment directly determine catalytic application. Especially for zeolite catalysts, the unique acidic and channel/cavity structure brought about enormous potential and excellent performance, which has been applied in a wide variety of commercial processes, for instance, ion-exchange, adsorptive separation/purification, fluid catalytic cracking (FCC), hydro-cracking (HC), methanol-to-hydrocarbons (MTH) and even biomass catalysis [1]. More than 235 molecular sieves with complex microenvironment embedding a variety of pore window and channel/cavity structures were discovered and defined as small-medium-large-pore zeolites. Among them, 8-membered ring (8-MR) cavity-type zeolites have been successfully used in separation, sorption and catalytic applications [1,2]. Remarkably, based on the shape-selectivity catalysis of the cavity-type zeolites, industry topologies have been explored on 8-MR zeolites and molecular sieves with cavity structure, such as selective catalytic reduction (SCR) of NO_x_ over Cu-SSZ-13 (CHA) [3] and methanol-to-olefins (MTO) process catalyzed on SAPO-34 (CHA) [2].

The representative application of 8-MR cavity-type zeolite is the methanol-to-olefins (MTO) reaction, a successful industrial process [2,4–6] for olefin production from non-oil resources, which can meet the main olefin demand without consuming crude oil. MTO process has received tremendous attention from both academia and industry, in which methanol, as a feedstock, can be produced from a variety of abundant sources, including coal [7], natural gas [8], biomass [9] and even carbon dioxide [10]. The successful application of MTO reaction, DMTO technology developed by Dalian Institute of Chemical Physics (DICP), Chinese Academy of Sciences [2,6], has enjoyed considerable success in economic income and technological innovation, launching a new era of sustainable olefin manufacturing from abundant non-oil resources since 2010. Since then, DICP has created the second and third generations of the DMTO process (DMTO-II and DMTO-III), and 31 commercial units with a total capacity of 20.25 Mt/a olefin have received licenses, of which 15 licenses have constructed the commercial-scale MTO plants with a total capacity of 8.36 Mt/a olefins [2,6].

SAPO-34, a CHA-type three-dimensional silicoaluminophosphate (SAPO) molecular sieve with a cavity dimension of 10.0 × 6.7 × 6.7 Å connected by an 8-membered ring pore window of 3.8 × 3.8 Å, was initially invented by researchers of the Union Carbide Corporation in 1984 [11]. The special confinement space of the cavity structure can provide accommodation for the active intermediates, and the 8-MR pore window can hinder entrance and diffusion of larger molecules, together with medium acidity and high thermal/hydrothermal stability, leading to the efficient production of extremely high light olefins [4–6,12–14]. In the late 1980s, the high catalytic performance and good regeneration stability of SAPO-34 in the MTO reaction was reported by DICP [15]. DMTO process was developed with a progressive amplification from the demonstration scale in 2006 to the world's first DMTO commercial unit conducted in 2010. Meanwhile, the manufacturing scale-up of the DMTO catalyst, mainly composed of SAPO-34, was successfully produced and delivered for DMTO commercial units.

**Figure 1.**
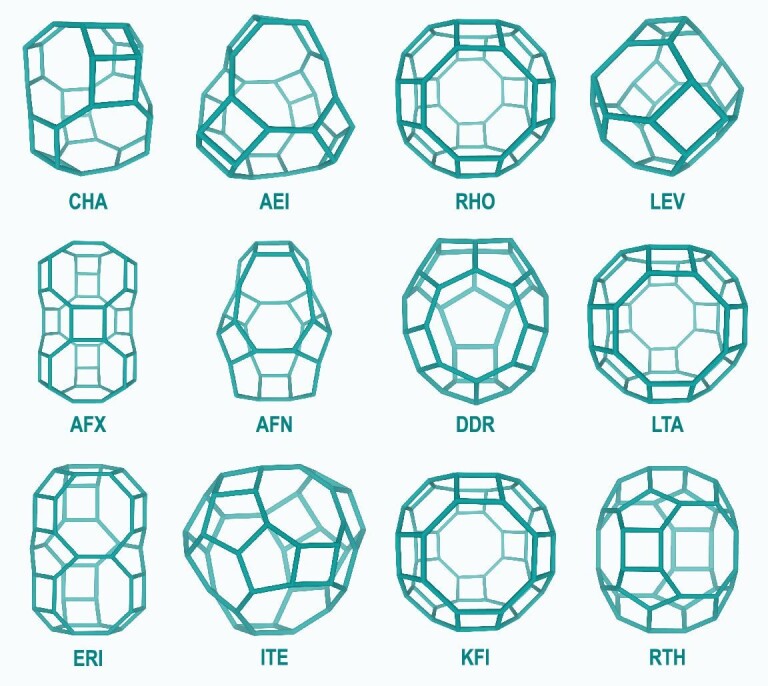
Cavity-type zeolites with 8-MR pore window.

Besides SAPO-34 with CHA structure used in industrial DMTO catalytic applications, other zeolites with 8-MR pore opening and cavity, such as AEI, RHO, LEV, AFX, AFN, DDR, LTA, ERI, ITE, KFI and RTH (Fig. 1), have also been regarded as potential and interesting candidates for methanol conversion [16–21]. Methanol conversion and product distribution during the MTO reaction is significantly affected by subtle variations in the cavity configuration of these 8-MR pore zeolites. For instance, the primary product is ethene over SAPO-35 (LEV) with the relatively small cavity [16]; higher selectivity for ethene and propene is demonstrated by SAPO-34 (CHA); propene and butene production is favored on SAPO-18 (AEI) and DNL-6 (RHO) with greater cavity spaces [16]; SAPO-14 (AFN) with an ultra-small cavity displays the highest propene selectivity [17]; SAPO-42 (LTA) with large 8-MR pore size and cavity exhibits higher selectivity to C_4_^+^ [21]; SAPO-56 with large AFX cavity favors ethene and propene generation [21], and so on. The distinct methanol conversion and product selectivity are directly related to acid-catalysis occurring in the microenvironment with the cavity structure and the 8-MR pore window. Shape selectivity of reactants, critical intermediates and products are also linked to the confined organic formation and molecular diffusion behaviors as the methanol conversion process is reacting and deactivating.

**Figure 2.**
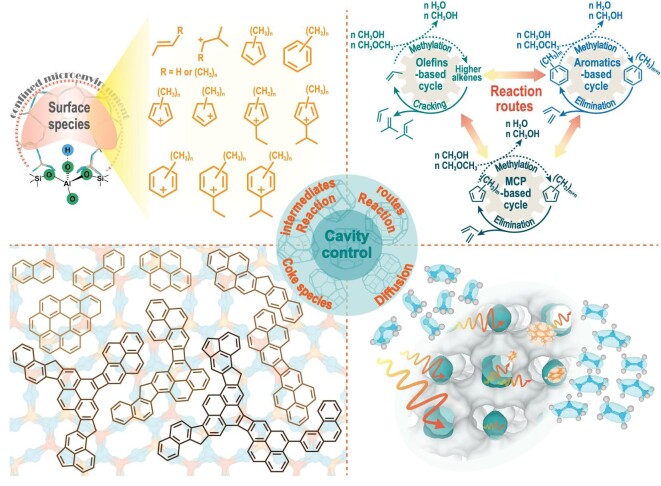
Cavity-controlled principle acted in the MTO reaction catalyzed over 8-MR and cavity-type zeolites.

Shape-selective catalysis is ubiquitous in the complete MTO reaction course [22,23], reflected by the formation of organic intermediates in the confined space [24], the special reaction mode for olefin product formation [25,26], deactivation with coke accommodation [27,28], reactant and products diffusion [29–32] and even the elementary steps in the reaction pathway of the complicated reaction network [18,33–35]. Notably, the microenvironment of zeolites embedding unique channel/cavity structure and acid properties has been taken as the dominant factor in the shape-selective catalysis process [34,35]. Especially for the 8-MR and cavity-type zeolites applied in the industry, the MTO reaction is significantly influenced by the cavity size and spatial confinement effect provided by the cavity-type catalyst. As shown in Fig. 2, the confined reaction intermediates and coke species in the zeolite cavity microenvironment, the preferential reaction routes in the complex reaction network, the catalyst deactivation and molecule diffusion join into cavity-controlled methanol conversion and drive the dynamic evolution of the MTO reaction [33,34]. During the process of methanol conversion, the ‘cavity-controlled principle’ is defined as a cage effect that originates from the unique cavity structure of zeolites, enabling precise control over the reaction intermediates, pathways, deactivation and diffusion. Owing to the complicated reaction system of the MTO reaction, the multiscale dynamic cross-talk behavior of catalyst material (coke)-reaction-diffusion in the methanol conversion process ultimately determines reaction performance with shape-selective catalysis [33].

Establishing the relationship between structure and reaction performance is critically significant to develop effective strategies on the design and control of the MTO process. In this review, we briefly summarize the most recent developments in the cavity-controlled reaction behavior in methanol conversion catalyzed on the representative 8-MR and cavity-type zeolites. Cavity-controlled methanol conversions reaction behavior, cavity-controlled formation of the hydrocarbon pool (HCP) species in the confined space and reaction pathway involving these crucial intermediates, cavity-controlled catalyst deactivation and diffusion behavior, and inspired controlled strategies are reviewed herein.

## CAVITY-CONTROLLED MTO REACTION BEHAVIOR

The successful application of SAPO-34 inspires the studies of MTO catalysis on the small-pore and cavity-type zeolite, and a large number of reports presented that the topology and cage-size of the cavity-type zeolite controls reaction activity, product selectivity and deactivation [16,19,20,25,27,36–38]. How the zeolite cavity affects methanol conversion and product selectivity and deactivation has been the essential concern of shape-selective catalysis within the MTO reaction, which is of significant importance to the achievement of a controlled strategy of methanol conversion and the desired product distribution in industrial applications.

Notably, the typical cavity-type zeolite featuring a wide range of cavity structures and an extremely close 8-MR pore opening, such as, SAPO-34 (CHA), SAPO-35 (LEV), DNL-6 (RHO), SAPO-18 (AEI), [16,19], SAPO-14 (AFN) [17], SAPO-56 (AFX) and SAPO-42 (LTA) [21] showed different product distribution and methanol conversion. The cavity size directly affects the product distribution. Over SAPO-34 (10.9 × 6.7 Å) of industrial application, higher selectivity for ethene and propene can be obtained in the methanol conversion process [2,5]. For the relatively small cavity molecular sieve, such as SAPO-35 with a small cavity (6.3 × 7.3 Å) and SAPO-14 with an ultra-small cavity (5.3 × 10.5 Å), ethene is the primary product over SAPO-35 [16] and the reaction over SAPO-14 exhibits the highest propene selectivity [17]. However, the production of propene and butene is facilitated by the greater cavity space of DNL-6 (11.4 × 11.4 Å) and SAPO-18 (12.7 × 11.6 Å) [16]. The 8-MR pore size, in addition to the cavity size, has a significant impact on the product distribution [21]. The large 8-MR pore size (4.1 × 4.1 Å) and cavity (11.4 × 11.4 Å) of SAPO-42 displays better selectivity to C_4_^+^, whereas the narrow 8-MR pore size (3.4 × 3.6 Å) and large AFX cavity (13.0 × 8.3 Å) in SAPO-56 favors ethene and propene formation from methanol conversion [21]. These findings imply that the reactant conversion and selective product formation in the MTO reaction are determined by the cavity type of the zeolite catalyst.

**Figure 3.**
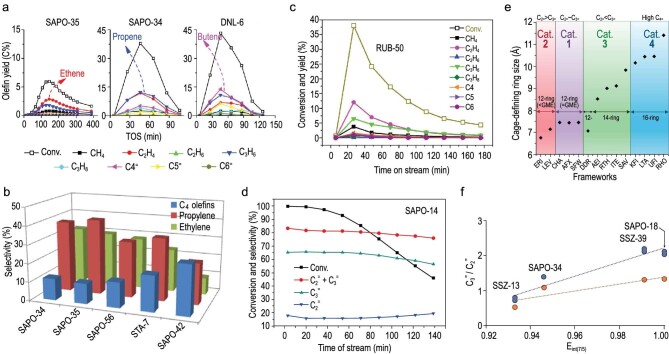
(a) Conversion and olefin yield over SAPO-35, SAPO-34 and DNL-6 in the MTO reaction. Adapted from ref. [16] with permission of the American Chemical Society. (b) Integral selectivity to C_2_-C_4_ olefins in the MTO process on SAPO-35, SAPO-56, STA-7, SAPO-42 and SAPO-34. Adapted from ref. [21] with permission of the Elsevier. (c) Methanol conversion and hydrocarbon product yields over H-RUB-50 catalyst. Adapted from ref. [38] with permission of the American Chemical Society. (d) Methanol conversion and product selectivity over SAPO-14. Adapted from ref. [17] with permission of the American Chemical Society. (e) Correlation chart of cage-defining ring size by cage structures and light olefin product distribution categories. Adapted from ref. [39] with permission of the American Chemical Society. (f) Relationship between measured C_3_^=^/C_2_^=^ ratio and E_int(7/5)_ parameter in small-pore cage-based zeolites and zeotypes. Adapted from ref. [40] with permission of the Wiley.

Due to shape-selective olefin production and the confined accommodation of the reactive intermediates in the cavity of the cavity-type zeolite, the influences of the spatial confinement and the size of the cavity on methanol conversion have attracted great attention [16,19,21,36]. The in-depth understanding on the varied MTO performance catalyzed on the small-pore zeolite with different cavities can help the establishment of the controlled strategy to methanol conversion. Bhawe and coworkers [36] studied the impacts of cage size of LEV, CHA and AFX type zeolites with 8-MR ring on methanol conversion to light olefins, and they found that ethene selectivity reduced as the cage size increased and the AFX material with a larger cage dimension (8.4  × 8.3 × 13.0 Å) exhibited the shortest reaction lifetime but the least quantity of coke deposition. The impact of cavity-type SAPO molecular sieves (SAPO-34, SAPO-18 and SAPO-35) on product distribution and coke generation in the MTO process were highlighted by Liu and coworkers [19]. The large-size cage of SAPO-34 and SAPO-18 favored propene and butene and the formation of confined organics of polycyclic aromatic species and poly-methyl–substituted adamantanes, whereas the predominant product over SAPO-35 was ethene, the aromatic and adamantane hydrocarbons were identified as the coke species. Subsequently, their group [16] investigated the organic reaction intermediates and their reactivity of confined species over three silicoaluminophosphate (SAPO) molecular sieves (SAPO-35, SAPO-34 and DNL-6) with different cavities and close 8-MR pore openings (Fig. 3a). They discovered that the size and reactivity of the confined intermediates are controlled by the cavity, which results in the difference in catalyst reactivity and product selectivity. Pinilla-Herrero and coworkers [21] compared the catalytic performance and deactivation in the MTO reaction on SAPO-35 (LEV), STA-7 (SAV), SAPO-56 (AFX), and SAPO-42 (LTA) with different topologies and cavity-windows (Fig. 3b). These differences in catalytic behaviors depended on the sizes of the SAPO cavities and the 8-MR that the zeolites of STA-7 and SAPO-42 with wider pore openings showed higher selectivity to C_4_^+^ hydrocarbons. Bulky aromatic coke species were not found on the SAPO-35 with small cavity, but SAPO-56 with a large cage favored the formation of heavy polyaromatic hydrocarbon coke compounds confined in the catalyst which caused the deactivation of SAPO-42. In addition, the relatively small cavity of RUB-50 (LEV) with higher ethene selectivity (Fig. 3c) was used as a representative small cavity zeolite to uncover the cavity-controlled MTO reaction system catalyzed by zeolites with strong host–guest interaction from the molecular level by Liu and coworkers [38]. Furthermore, recently, Liu and coworkers [17] hydrothermally synthesized an ultra-small cage SAPO-14 molecular sieve (AFN topology) (5.3 × 10.5 Å), which showed the highest selectivity for propene (Fig. 3d), providing a protocol for the MTO reaction regulation to realize high propene selectivity.

Zeolite catalysts with various cavity structures mediate the reaction performance by changing the formation of crucial intermediates, olefin precursors and their involvement route in the complex reaction network based on the strong host–guest interaction of the supramolecular system, which jointly affect the MTO reaction's product selectivity. The cavity environment directly controls product distribution of the MTO reaction over cavity-type and small-pore zeolite and zeotype materials [34,38–40]. Liu and coworkers [38] established the definitive correlation between the catalyst's cavity structure and product selectivity by assessment of all the elementary steps of the complete catalytic cycles at the molecular level, and found that predominant formation of olefin products depended on the generation of HCP species, the formation of the olefin precursor and the reaction route together were mediated by different cavity-type zeolite or zeotype catalysts. Davis and coworkers [39] proposed the concept of the cage-defining ring size to clarify the structure–property relationship by combining the zeolite structural indicator with olefin product distribution. The sizes of intermediate polymethyl aromatic compounds are constrained by the size of the cage-defining ring, correlating to light olefin product distributions (Fig. 3e). Besides, Corma and coworkers [40] found that the selectivity ratio of propylene to ethylene (C_3_^=^/C_2_^=^) for different zeolites (Fig. 3f) links to an interaction energy ratio E_int(7/5)_ parameter (the relative stabilization of heptamethylbenzenium cation (7MB^+^, priority to propene) and pentamethylbenzenium cation (5MB^+^, priority to ethene)) and proposed that the stability of critical intermediates originated from the confinement effect of the cavity structure that works as a key factor that controls product distribution, mediating MTO product selectivity. Understanding and describing the link between structure and product give directions to realize the formation of the desired product in the methanol conversion. The cavity-controlled MTO reaction behavior is a dynamic ‘catalyst material-reaction-diffusion’ crosstalk behavior. All these factors working together lead to the cavity-controlled reaction behaviors and principles, which are demonstrated by the cavity-controlled formation of reaction intermediates, cavity-controlled reaction route with the involvement of these intermediates, cavity-controlled reactant and products diffusion, and cavity-controlled catalyst deactivation in the microenvironment of the 8-MR and cavity-type zeolites.

## CAVITY-CONTROLLED REACTION INTERMEDIATES AND REACTION ROUTES

Cavity environment zeolite catalysis has been considered as crucial for the MTO reaction, which makes the reaction intermediates and reaction pathways complex and drives the dynamic evolution of methanol conversion due to the host–guest interaction in this special catalytic microenvironment of 8-MR and cavity-type zeolite materials [41–44]. This unique cavity microenvironment covers multiple interactions originating from the cavity space limitation, electrostatic state and stabilization, active center and geometry mobility of the reactive intermediates [43]. During the dynamic course of the MTO reaction, zeolite microenvironment with the cavity-type structure, directly controls the production of the active organic species, the C-C bond assembly, the establishment of an autocatalytic hypercyclic network and the product formation [35].

Over 40 years’ developments of MTO chemistry and industry have gradually deepened our knowledge on methanol conversion. Starting from the initial surface species of carbocation, radical species, oxonium ylide, carbene and methane-formaldehyde admixture [45–51] as reaction intermediates for methanol conversion, 20 different direct reaction mechanisms were proposed for initial C-C bond production during the early stage. Via the olefins as autocatalytic species [52,53] and co-feeding aromatics species accelerating methanol conversion [54], the proposal of the hydrocarbon pool concept [55,56] explains olefin formation in the steady-state stage. Simultaneously, inspired by this observation, identification of HCP species and their function in the catalytic reaction network, the fundamental research development of the MTO reaction obtained enormous progress and in-depth understanding [57–64]. Aromatics-based routes (side-chain and paring routes) [65], alkenes-based routes [53], dual-cycle [66] and cyclopentadienes-based cycle [18] proposed one after another, give more refined and rational catalytic cycles and networks, presenting complex and cooperative methanol conversion. The dynamic reaction network, running from initiation to the building of incipient autocatalysts, maintained by a hypercyclic network and rapid extinction of autocatalysis, exhibits dynamically evolving molecular pathways and reaction networks with multi-reaction route interaction [35].

MTO zeolite catalysis process with multiple reaction stages and complex reaction network, behaves in the dynamic and complex way in the zeolite confined microenvironment [35]. Especially, for the zeolite confinement microenvironment with small pore window and cavity-type structure, cavity not only controls the generation of HCP species but also mediates the reaction route with the participation of these critical intermediates in the complex reaction network that, ultimately, determines MTO reaction performance and product selectivity [16,38].

### Cavity-controlled formation of hydrocarbon pool species

When the initial olefins are formed, the HCP species proposed by Dahl and Kolboe *et al.* [55,56], starts to accumulate and methanol conversion exhibits an autocatalytic character [67,68]. This hydrocarbon pool concept prompted the development of ‘hydrocarbon pool mechanism’ for the efficient reaction stage of methanol conversion, in which light olefins are eventually produced via an indirect pathway with the participation of HCP species, a kind of supramolecular active center confined in the cages or channels of the zeolite. Subsequently, the observation and identification [57–64] of HCP species have become the focus of research, leading to complex networks consisting of a variety of detailed reaction routes [18,35,53,65,66]. In recent work, an autocatalytic reaction network [35] has been established, in which autocatalysis is driven by a hypercycle with the one ‘selfish’ autocatalysis cycle interlinked with three cross-catalysis cycles in methanol conversion. This dynamic process of C-C bond assembly in methanol conversion is directly mediated by the supramolecular active centers. HCP species, olefinic, cyclopentadiene and aromatic species, and the zeolite microenvironments (acid center and its surrounding confined space channel and/or cavity) work together to guide methanol conversion as the true active center.

Starting from the proposed hydrocarbon pool concept, polymethylbenzenium cation, polymethylcyclopentenyl cation and their corresponding neutral species, and their co-catalytic function in the MTO reaction over different zeolites received enormous attention. Most interestingly, in the early studies, it is found that the zeolite channel/cavity brings about the difference of the HCP species and their involvement mode in olefin formation. For the H-ZSM-5 zeolite with intersectional channels (5.1 × 5.4 Å, 5.4 × 5.6 Å), in the co-feeding reaction of ^13^C-methanol and ^12^C-benzene or toluene, 50%–75% ^13^C atoms appearing in ethene and propene and ^13^C atoms incorporation in polymethylbenzenes provided direct evidence that the polymethylbenzenes are involved in the olefin production process [69]. Subsequently, on the H-Beta with wider channels (7.6 × 6.4 Å and 5.5 × 5.5 Å) [70,71], the addition of polymethylbenzenes can significantly accelerate methanol conversion, proving the reactivity of HCP species. Especially, over H-SAPO-34 with cavity structure of 6.7 × 10.9 Å, Song, Arstad and Kolboe *et al.* [57–59] confirmed polymethylbenzenes as the prominent reactive species and their co-catalytic function in methanol conversion via solid nuclear magnetic resonance and pre-deposition of organics, and established the link between olefin production and the methyl group number of benzenes. Based on the host–guest interaction between the confined organic HCP species and the inorganic molecular sieve, a supramolecular system was proposed by Haw and coworkers [57] that the molecular sieve characteristics (topology, element composition and acidity) and the reactivity of the HCP species contained in the molecular sieve work together for specific performances in methanol conversion.

**Figure 4.**
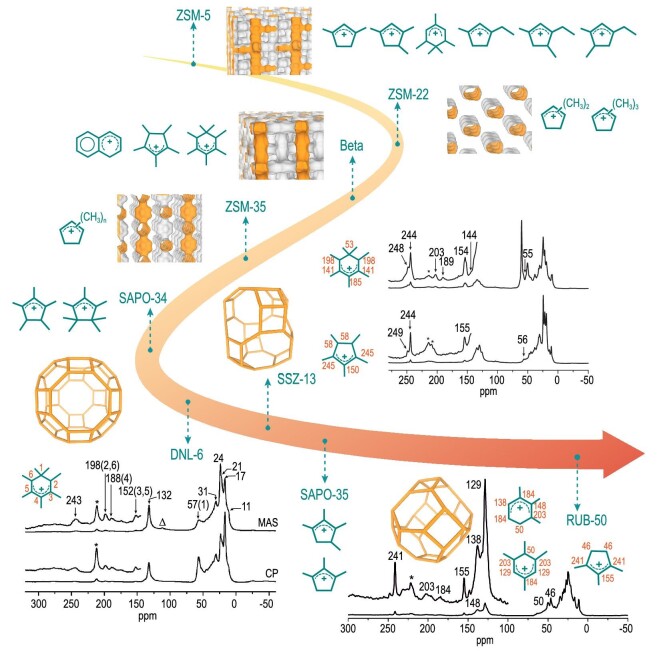
Series of carbenium ions observed in different zeolites by solid-state NMR spectroscopy during methanol conversion. Adapted from refs [38,64,80] with permission of the American Chemical Society and Wiley.

Notably, carbenium ion intermediates [60,61,72] play significant roles in methanol conversion, and many studies have been devoted to capture and identify the critical carbenium ion intermediates in different zeolites. However, due to their higher reactivity, instability and low concentrations, the observation and identification of carbocations has always been a huge challenge, therefore, gas chromatography-mass spectrometry (GC-MS), ^13^C-switch experiment and density functional theory (DFT) calculations [35,62,73] and solid state NMR techniques, including ^13^C NMR [35,60–62,73–75] and dynamic nuclear polarization (DNP) magic angle spinning nuclear magnetic resonance [42,76] were used to detect these critical intermediates. HCP carbenium ion intermediates confined in different cavity-type zeolites as shown in Fig. 4 were discovered and their formation was correlated to the zeolite topology, framework composition, strength of acid sites, pore window and reaction condition. Using multi technologies, such as the a pulse-quench catalytic reactor combined with *in situ*^13^C NMR measurements [62], solid state nuclear magnetic resonance (ssNMR) combined with gas chromatography-mass spectroscopy [73] and two dimensional ^13^C-^13^C refocused INADEQUATE spectrum [42,76], series of carbenium ions, consisting of di/trimethylcyclopentenyl cations, pentamethylbenzenium cations, ethylcyclopentenyl cations and butylcyclopentadienium cations [62,73–75] were captured in H-ZSM-5 with three-dimensional (3D) channel system, consisting of a straight channel of 5.1 × 5.5 Å intersected by a sinusoidal channel of 5.3 × 5.6 Å. On the H-Beta with larger size channels (7.6 × 6.4 Å and 5.5  × 5.5 Å), Liu and coworkers [77] observed heptamethylbenzenium and pentamethyl cyclopentenyl cation and Blanc and coworkers [42] detected five, 6-membered ring carbocations and methylnaphthalenium ions by DNP enhanced multinuclear NMR spectroscopy on different microstructural Beta-zeolites. Yet for ZSM-22 [78] with one-dimensional straight 10-membered ring channel (5.7 × 4.6 Å) and ZSM-35 [79] with two-dimensional textural structure with intersection of 10-membered ring channel of 5.4 × 4.2 Å and 8-membered ring channel of 4.8 × 3.5 Å, only cyclopentenyl cations with different methyl groups were found.

To reveal the difference of the product selectivity on the 8-MR pore and cavity zeolite, cavity structure was found to be of great influence on the formation of the reaction intermediates. As shown in Fig. 4, in 2012, first, under real working conditions, on a recently created large-cavity SAPO-type molecular sieve (DNL-6, 11.4 × 11.4 Å), Liu and coworkers [64] observed heptamethylbenzenium cation (heptaMB^+^) during methanol conversion, which mediates olefin formation via the side-chain cycle of the hydrocarbon pool mechanism. Subsequently, heptamethylbenzenium and pentamethylcyclopentenyl cations were found to be formed and confined in the H-SSZ-13 [80] under real MTO reaction conditions, and the generation of these carbenium ions is a result of acid catalysis in the confined microenvironment [77,80]. Besides, for the SAPO-type molecular sieve with CHA (H-SAPO-34), LEV (H-SAPO-35) and RHO (DNL-6) topology, the size and reactivity of carbenium ions were controlled by cavity size, causing the differences in MTO reactivity and product selectivity. Recently, a representative zeolite H-RUB-50 with a small cavity (6.3 × 7.3 Å) was employed as an example of the MTO reaction system's host–guest interaction [38], and trimethylcyclopentenyl and tri/tetramethylbenzenium cations with less methyl groups were captured as critical active species, differing from heptaMB^+^ and pentaMCP^+^ captured over SSZ-13, SAPO-34 and DNL-6, and the carbenium ions matching well with the zeolite cavity structure led to the high reactivity of these intermediates.

These HCP intermediates are associated with their zeolite microenvironments with different cavity structure, as the true supramolecular active centers, wherein a large cavity enables the production of massive HCP carbenium ion intermediates and less methyl-groups–substituted HCP carbenium ion intermediates are more likely to be produced in small cavity zeolites. In this supramolecular microenvironment catalysis, the explicit difference in the identification of the confined organic intermediates for various catalysts shows the impact of cavity-controlled intermediate production in the MTO reaction system catalyzed on zeolite with strong host–guest interaction. Based on the knowledge of the host–guest interaction of the MTO reaction system, it can be predicted that methanol conversion route and the selective production of desired olefins would be adjustable by altering the catalytic environment with cavity-type structure.

### Cavity-controlled reaction routes

Diversified zeolite microenvironments (with channel and/or cavity structure in combination with acid center) accommodating complex HCP species brings out a diverse reaction route and product generation, corresponding to the MTO performances variation with the catalysts used. Zeolite steric constraints mediated critical intermediates and the preponderant reaction route in the complex reaction network served as the emphasis for shape-selective catalysis in both MTH chemistry and industry. Beside the capture, identification and evolution of the HCP species, their participation in the catalytic reaction cycle and the corresponding reaction route establishments to form the complex reaction network under the confined zeolite microenvironments have attracted great research effort [16,38,57,64,66,80–84].

**Figure 5.**
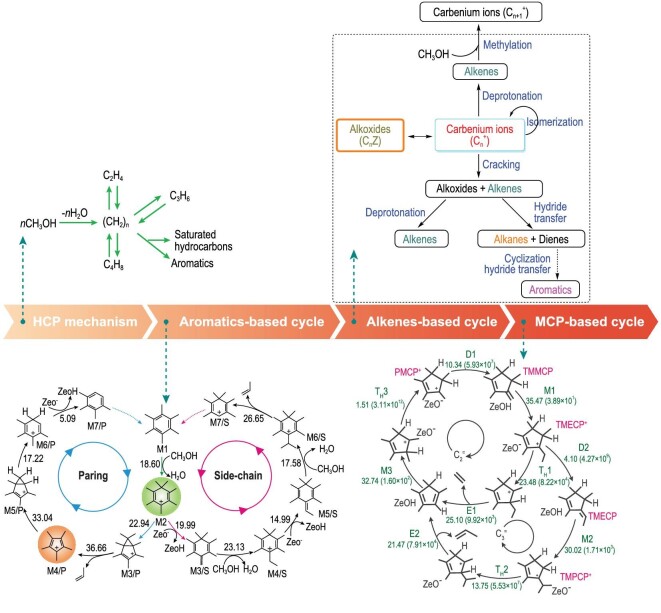
The evolution of the MTO mechanism with the involvement of the critical intermediates over cavity-type zeolite. Adapted from refs [18,55,56,80,85] with permission of the Elsevier, American Chemical Society and Wiley.

Based on the capture and identification of the HCP species in the confined zeolite environment, hydrocarbon pool mechanisms including multiple catalytic cycles (Fig. 5) such as aromatics-based cycle (side-chain and paring cycle), alkenes-based cycle and cyclopentadienes-based cycle were proven to run together for the C-C bond assembly via HCP pathways in the MTO reaction system [18,35]. Meanwhile, these critical intermediates are interrelated through interconversion among themselves. Olefin products tend to be the preferred mechanistic cycles that are mediated by the involved intermediates on the zeolite catalysts, leading to differences of the product generation and distribution. The dual-cycle mechanism, proved by the ^12^C/^13^C-methanol switch experiments over H-ZSM-5, revealed that ethene generation via the aromatics-based cycle with the involvement of the lower methylbenzenes varied from other higher olefins formation, C_3_^+^ olefins being generated via olefin methylation and cracking steps (the alkenes-based cycle) [66]. A complete route of paring cycle for the formation of isobutene from methanol was suggested by Haw and coworkers [84]. Also, Song [57] and Wang [83] suggested that polymethylbenzenes with more methyl groups produced mainly propene and higher olefins, whereas methylbenzenes with less methyl groups favored the formation of ethene. In addition, Bhan and coworkers [82] pointed out that ethene is a product unique to aromatics-based dealkylation pathway, butene originates from both alkenes-based methylation and ß-scission and/or aromatics dealkylation, and pentene and higher olefins are the product of olefins methylation events. Different topologies and different acidity of zeolite catalysts would likely lead to differences in the preference of catalytic cycles and olefin generation.

When the methanol reacts on the bulky-sized zeolite, such as SAPO-34 (CHA), SAPO-18 (AEI) and DNL-6 (RHO), heptmethylbenzenium and pentamethylcyclopenyl cations, are confirmed as the most critical active intermediates, while for the small-sized cavity zeolite (LEV), relatively small-sized intermediates, such as tetramethylbenzenium and trimethylcyclopentadienyl cations, participate in methanol conversion [16]. Most importantly, it is critical to understand product selectivity from the preferred reaction pathway with the participation of these intermediates for different olefin product formations. For example, with the participation of pentaMCP^+^ and heptaMB^+^ in H-SSZ-13, the activation energies of rate-determining step for the paring (36.66 kcal mol^−1^) and the side-chain methylation (26.65 kcal mol^−1^) mechanism confirmed that the side-chain methylation mechanism predominates over H-SSZ-13 zeolite in the MTO reaction [80]. H-RUB-50 zeolite, with an 8-MR and small cavity-type zeolite presenting higher ethene selectivity, was employed to clarify the cavity-controlled principle of the MTO reaction [38]. Comparing the side-chain methylation mechanism with the paring mechanism revealed that the side-chain route is the favored reaction pathway for olefin production. Following that, according to the overall assessment of the catalytic cycle, combining the difference of energy span of the entire reaction pathway for the production of ethene and propene, and the formation of the ethene and propene precursors, a direct theoretical demonstration of the product selectivity and the preferred formation of ethene are obtained. Liu and coworkers found, with the help of ssNMR and GC-MS measurement experiments, that most of the ^13^C atoms embedded into the methyl group rather than the benzene ring in methanol-reacted DNL-6 [64,81], suggesting that the primary mechanism for olefin formation during MTO conversion over DNL-6 follows the side-chain methylation route and expansion of the cyclic intermediate in the paring route is difficult to achieve for methanol conversion. For the ultra-small cage pore SAPO-14 molecular sieve (AFN topology), by combining the analyses of ^12^C/^13^C-methanol isotopic switch experiments and reaction-diffusion simulations, the alkenes-based reaction pathway and the diffusion restriction of smaller 8-MR pore openings are responsible for propene formation with higher selectivity [17]. These results revealed the cavity-controlled reaction intermediates and reaction route with the involvement of the confined intermediates in the cavity, which are of benefit to the comprehensive understanding of the product selectivity and provide reliable strategies to regulate MTO conversion by designing and synthesizing molecular sieve catalysts with shape selectivity.

## CAVITY-CONTROLLED COKE FORMATION AND CATALYST DEACTIVATION

With the proceeding of methanol reaction and olefin formation, HCP species (alkenes, cyclopentadienes and aromatics) start to accumulate in the channel/cavity of zeolites. The fate of HCP species goes in two directions, one is as active intermediate that can be methylated with methanol to achieve propagation of the C-C bond and then eliminate to light olefins [18]; the other is that they can transform to the polycyclic aromatic hydrocarbons (PAHs) species as coke species, blocking the pore or covering the acid sites and resulting in deactivation [86,87]. The production of coke species is critical for the deactivation mechanism and how the HCP species transform to coke precursors such as oxygen-containing coke species, dihydrotrimethylnaphthalene, dihydro-1,5,6-trimethly-1H-indene, tetrahydro-1,8-dimethylnaphthalene, 1,2-dimethyl-3-(2-butenyl)benzene [28] and further to PAHs with cage-passing growth [86]. Two industrial molecular sieves, SAPO-34 (8-MR CHA cavity topology) and ZSM-5 (10-membered ring channel MFI topology), display various deactivation patterns during the conversion of methanol. In the next section, we briefly review the deactivation behaviors on SAPO-34 which reflect the cavity-controlled deactivation over MTO reaction catalyzed over 8-MR and cavity-type zeolites.

### Relating the deactivation mode to coke formation in cavity of H-SAPO-34

8-MR and cavity structure of H-SAPO-34, which has an excellent catalytic performance and light olefin selectivity, has drawn increased interest from both fundamental research and practical applications. Due to inevitable deactivation with coke deposition in the cavity, DMTO process employed continuous cyclic reaction-regeneration technology [2]. For the complex MTO reaction system, to fully understand the deactivation process, it is crucial to identify the relationships between reaction and coke deposition, coke deposition and diffusion, and coke deposition and deactivation [87].

**Figure 6.**
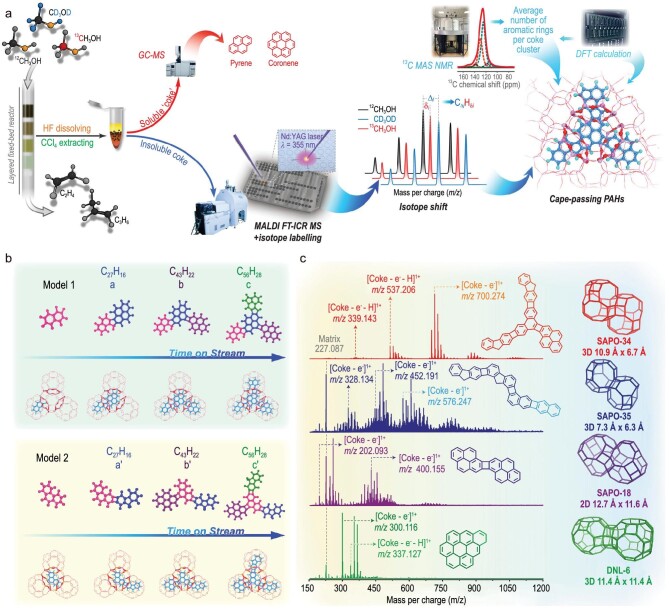
(a) Schematic illustration of an integrated strategy for deciphering full-spectrum PAHs. The proposed analytic method for heavier PAHs by combining the advanced MALDI FT-ICR MS with ^13^C magic-angle spinning nuclear magnetic resonance (MAS NMR) spectroscopy and density functional theory (DFT) calculations. (b) The conceivable molecular structures of PAHs in SAPO-34 with optimized configuration. (c) PAHs analysis of cage-structured molecular sieves. Adapted from ref. [86] with permission of the Nature.

The understanding of the deactivation mechanism of the zeolite-catalyzed MTO reaction is being advanced by a number of fresh insights. In 2012, newly discovered species of non-aromatic hydrocarbons [88] (diamondoid compounds, especially methyl-substituted adamantanes) as the confined coke species were discovered to be the deactivating species in the MTO reaction at low reaction temperatures of 300–325°C, demonstrating that cavity structure may offer a suitable catalytic environment for the formation of H-saturated deactivation species with multiple rings. Subsequently, the evolution of retained species from methylbenzenes to methylnaphthalenes during the deactivation process were revealed [28], according to the first capture of three important intermediate species, dihydro-1,5,6-trimethly-1H-indene, tetrahydro-1,8-dimethylnaphthalene, 1,2-dimethyl-3-(2-butenyl) benzene and their possible isomers. During the whole MTO process, the transformation from polymethylbenzenes to methylnaphthalenes was crucial in the deactivation of the MTO reaction. Precise routes for the transformation of retained methylbenzenes to methylnaphthalenes were provided. Furthermore, with the aid of confocal fluorescence microscopy and HP ^129^Xe NMR, an irregular spatial distribution with a yolk-shell–like coke location was found in SAPO-34 [89]. Pulsed field gradient (PFG) NMR and diffuse DRIFTS were used to track the diffusion coefficients and loss of accessibility of acidic sites in the coke catalysts during the MTO reaction, respectively, implying that coke accumulation in the cavity severely prevents the mass transfer of reactants and product molecules and results in the SAPO-34 catalyst's deactivation. Recently, a cage-passing growth mechanism has been proposed and confirmed to be universal in 8-MR and cage-structured molecular sieves by a comprehensive analysis approach combining the Matrix-Assisted Laser Desorption/Ionization Fourier-transform Ion Cyclotron Resonance Mass Spectrometry (MALDI FT-ICR MS) combined with isotope labeling (Fig. 6a) [86]. It is possible to investigate bulky PAHs molecules without destroying or fragmenting the sample by using the MALDI soft ionization technique. The detected coke species have a mass distribution of 300 to 1200 Da (three to four ring aromatic mass) exceeding the mass maximum sized molecule (pyrene) accommodated in one CHA cage, illustrating that the bulky PAHs occupy numerous proximal cages passing through the adjacent 8-membered ring windows. The basic coking units/clustering seed, three- to four-ring aromatic compounds, are first produced in single cages, and, eventually, they gradually form cross-links with those in the nearby cages (Fig. 6b). The complete evolution pathway of coke deposition was provided: in the SAPO-34 molecular sieve cage, three- to four-ring coke precursors originated from the active HCP compounds via ring expansion reactions, following which a cage-passing growth mode promotes the production of cross-linked multi-core PAHs, representing the cage structure inducing the construction of the passing coke species and PAHs accommodation in the cavities around neighborhood.

### Cavity-controlled coke formation

Among 8-MR and cavity-type zeolites, such as CHA, AEI, DDR, RHO, LEV and AFX, the potential candidates for the MTO process, the reaction behaviors including PAHs coke formation in the cavity-type zeolites can be significantly affected by slight variations in their framework topology [16–21]. Establishment of the relationship between deactivation and topology would be of benefit to the coke-related strategy proposal to prolong the catalyst lifespan, improve the shape selectivity by pre-coking and regenerate the industrial catalyst in more sustainable ways.

The difference of the molecule sieve topology, especially for the cavity-type zeolite with 8-MR, directly leads to different coke species deposition and catalyst deactivation due to the cavity-controlled effect in the zeolite microenvironment. Hong and coworkers [27] investigated the deactivation of the ERI (UZM-12), CHA (SAPO-34), UFI (UZM-5) and LTA (UZM-9) with cavity structure and 8-MR pore opening. They found that catalysts for ERI, LTA and UFI deactivated quickly, but the CHA catalyst showed a stable conversion, ascribing quick deactivation to the blockage of 8-MR pores with the accommodation of larger polycyclic aromatics. The differences of the coke generation species over SAPO-34, SAPO-18 and SAPO-35 were revealed by Liu and co-workers [19]. The monocyclic and bicyclic aromatic species were generated among all these cavity-type catalysts, whereas polycyclic aromatic hydrocarbons appear in the deactivated SAPO-34 and SAPO-18 with larger cavity structure. The small LEV cavity structure depresses the formation of the larger PAHs species, while the larger CHA and AEI cavity structure provides sufficient space to accommodate polycyclic aromatic compounds.

**Figure 7.**
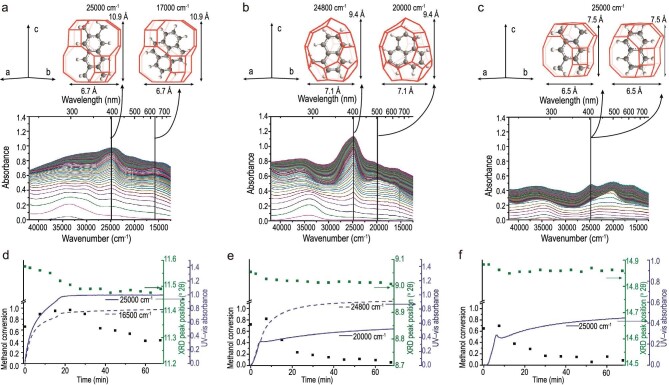
Operando UV-vis spectra during methanol conversion over the (a) CHA, (b) DDR, (c) LEV catalysts. Hydrocarbon species corresponding to the UV-vis absorbance bands, i.e. (a) tetramethylnaphthalene and pyrene, (b) 1-methylnaphthalene and phenalene, (c) tetramethylbenzene and naphthalene, are compared to the size of the (a) CHA, (b) DDR, (c) LEV cage. Combination plot for (d) CHA, (e) DDR, (f) LEV cage, showing the relation between methanol conversion (left-axis), corresponding lattice expansion (XRD peak position, first right y-axis), and amount of retained hydrocarbon species. Adapted from ref. [90] with permission of the American Chemical Society.

Multi methods, such as UV-vis spectroscopy, IR, Raman spectroscopy, confocal fluorescence microscopy, Laser Desorption/Ionization-Time of Flight Mass Spectrometry (LDI-TOF MS) and MALDI FT-ICR MS MALDI FT-ICR mass spectrometry were used to identify the structure of PAHs [86,90,91]. Using operando UV-vis spectroscopy, Weckhuysen and coworkers [91] explored the nature and evolution of confined organics in the MTO process on 8-MR cavity-type zeolites (CHA, DDR and LEV) and confirmed that hydrocarbon accumulation is responsible for small-pore zeolite deactivation. For these small-pore zeolites, slight variations in size and shape of the cages would lead to great difference in the amount and nature of coke species during the MTO reaction. Different hydrocarbon species, methylated naphthalene and pyrene in CHA, methylated benzene and naphthalene in LEV, and 1-methylnaphthalene and phenalene in DDR were confirmed. Based on combining with operando X-ray Diffraction (XRD) spectroscopy, lattice expansion of three different small-pore zeolite frameworks (CHA, DDR and LEV, as shown in Fig. 7) is assigned to the position of hydrocarbon compounds in the cavity of the zeolites [90]. In a recent study [86], the cage-passing growth mechanism of PAHs coke species was proposed as a universal mode of coke formation over 8-MR and cavity-type catalysts with varied topology (SAPO-35, SAPO-18, DNL-6). Different coke units exhibit different cavity structures, biphenyl mode between the coke units of naphthalene and fluorene in the small cage SAPO-35 (LEV, 7.3 × 6.3 Å), pyrene coke unit in the large cage SAPO-18 (AEI, 12.7 × 11.6 Å), coronene without a cage-passing event occurred in the RHO cavity of DNL-6 with the largest dimension (12.7 × 11.6 Å for the former and 11.4 × 11.4 Å for the latter) (Fig. 6c). These works uncover shape-selective catalysis for coke formation, in which the chemical nature, formation and evolution of coke species are cavity-controlled in terms of cavity structure.

## CAVITY-CONTROLLED DIFFUSION

Methanol-to-olefins (MTO) reaction catalyzed by molecular sieves with 8-MR and cavity-type structures is governed by cavity control, which controls the generation and stabilization of the HCP species, determining the predominant reaction route and olefin product network during methanol conversion. The microenvironment consisting of acidity and cavity with an 8-MR pore window has a significant impact on the diffusion behavior of the reactants, the hydrocarbons and the products. Due to diffusion limitations of higher hydrocarbon products over the small 8-MR pore opening of cavity-type zeolites, extremely high selectivity toward light olefins is achieved. Therefore, in the zeolite-catalyzed MTO process, the guest molecular diffusion in the constrained environments is critical for high reaction activity and selective product generation [37]. A complete description linking the reaction and diffusion is crucial for the proposed strategy of reaction control, which is the most critical issue for important catalytic applications. To fully understand the diffusion mechanism and advance shape-selective strategies for effective catalytic processes, it is crucial to uncover the host–guest interaction, the diffusion pathway, and the quantifying of the confinement effect of different cavity structures on diffusion behavior, which is essential in optimizing the diffusion-controlled reaction process and product regulation of the MTO process.

### Cavity-controlled diffusion behavior in cavity-type zeolites

**Figure 8.**
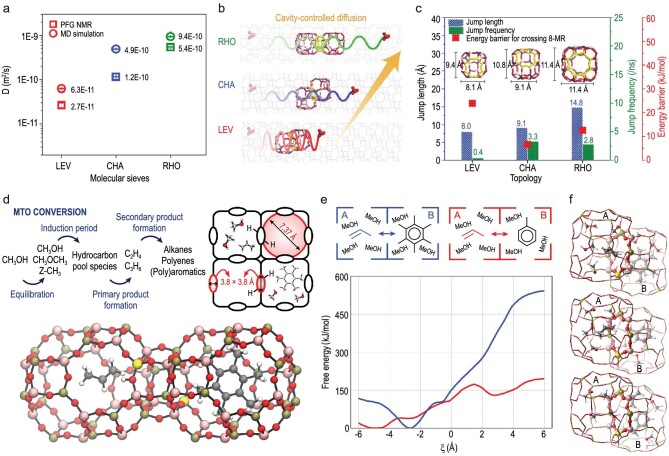
(a) The experimental (PFG NMR) and simulated (MD) self-diffusion coefficients of methane in LEV, CHA and RHO at the loading of two molecules per cavity at 298 K. (b) The cavity-controlled diffusion behavior on the cavity-type zeolite. (c) The average jump frequency jump length and diffusion energy barrier of methane in LEV, CHA and RHO extracted from diffusion trajectories via the CTRW coarse-graining method and the corresponding energy barrier for crossing 8-MR of methane in the inter-cavity hopping process. Adapted from ref. [37] with permission of the Elsevier. (d) Scheme of reaction and two adjacent cages containing HP species in the H-SAPO-34 pore system with large cages connected via 8-ring windows. (e) Free energy profile for propene diffusion through an 8-ring type 1 of H-SAPO-34 at 650 K from AI-US simulations. Cage B contains a HCP species (hexamethylbenzene (HMB) or toluene (TOL)). (f) Snapshots from the regular AI-MD simulations at 650 K of the local minima on the free energy surface corresponding to propene adsorbed in cage A and hexamethylbenzene in cage B, propene adsorbed in cage A and toluene in cage B, and propene and toluene coadsorbed in cage B . Adapted from ref. [29] with permission of the American Chemical Society.

In order to explore the confinement effect on the diffusion of guest molecules, cavity-type molecular sieves with closely spaced 8-MR windows and various cavities have been selected to illustrate the diffusion behavior in the cavity structure catalyst. Ghysels and coworkers [30] suggested the ring-dependent diffusion behavior of ethene and propene in the MTO process affected by the composition, acidity, and flexibility of the molecular sieves of AEI, CHA AFX, topologies relevant for the MTO process by the aid of molecular dynamics simulations techniques. A new descriptor of the accessible window area representing the free space available for product molecules was proposed to establish a link between molecule size and diffusion behavior. Using an advanced PFG NMR technique, combined with molecular dynamics (MD) simulations, Liu and coworkers [37] explored the diffusion behavior of alkanes (methane, ethane and propane) in three 8-MR cavity-type molecular sieves (LEV, CHA and RHO), and found that the diffusion rate order of methane on DNL-6 (RHO, 40 × 10^−10^ m^2^/s) > SAPO-34 (CHA, 13 × 10^−10^ m^2^/s) > SAPO-35 (LEV, 4.2 × 10^−10^ m^2^/s) are determined by the cavity structure of the 8-MR molecular sieves (Fig. 8a and b). Furthermore, the quantitative correlation between diffusion behavior with cavity hopping behavior characteristic of the jump frequency and jump length in the cavity-type molecular sieves were established via the continuous-time random-walk coarse-graining method. High jump frequency, long jump length and lowest diffusion activation energy in large *lta* cage (RHO) environment correspond to the fastest methane diffusion, while in the small-cage LEV structure, lowest diffusion coefficient is accompanied by low jump frequency and short jump length, which is ascribed to the confinement or limitation of the cavity structure and the distance of adjacent cavities (Fig. 8c). These cavity-controlled diffusion behaviors, combined with previous studies of the cavity-controlled reaction intermediates and catalytic route, together explain the product distribution differences: RHO-SAPO favors butene production, ethene is formed over LEV-SAPO, and propene is the main product over CHA-SAPO under reaction conditions. Subsequently, ^129^Xe NMR spectroscopy using xenon atom as sensitive probe molecular and PFG NMR spectroscopy, reveal the function of the *lta* cage and double 8-membered rings (D8R) of DNL-6 molecular sieves (RHO) on dynamic adsorption and diffusion behavior [92]. Simultaneously, direct tracking MTO reaction [32] and the assessment of diffusivity has been realized by a home-made pseudo-gas chromatography method under actual MTO reaction conditions and the extracted intracrystalline and intercrystalline diffusion behavior provided the quantitative and dynamic evolution of the reactant and product shape selectivity.

### Diffusion in acidic cavity with confined organic species

Understanding product diffusion in the dynamic confined environment of zeolites with reactants and HCP compounds under real MTO reaction conditions, combined with the production formation mechanism, will provide a complete explanation of the product selectivity. The self-diffusivity of ethene and ethane was reported to systematically decrease with increasing MTO reaction time by Dai and coworkers [93], using the PFG NMR, which was ascribed to a growing hindrance by the HCP compounds on molecular diffusion. The ratio of the self-diffusivities of ethene and ethane, was used to evaluate the diffusion selectivity and the difference of the product selectivity. The improvement of the diffusion selectivity is of benefit to the smaller olefin when the organic species is constrained in the SAPO-34 cavity with increasing MTO reaction time. Besides, considering the existence of the Brønsted acid sites, HCP compounds, and larger methanol loadings, Van Speybroeck and coworkers [29] studied the diffusion of light olefins over H-SAPO-34 with a complex dynamic molecular environment during the MTO reaction. With the aid of enhanced sampling molecular dynamics techniques, they found that Brønsted acid sites located at the 8-MR would help to decrease the diffusion barrier due to π-H interactions between the Brønsted acid site and the olefin. Notably, the existence of HCP species hinders olefin diffusivity, as shown in Fig. 8d and e, the barrier for propene is much higher with hexamethylbenzene species confined in cage A than that in the case of toluene. In recent work, Cnudde and coworkers [31] investigated the impact of Brønsted acid sites on alkene and alkane diffusivity, finding that increasing the acid site density can enhance alkene diffusion (the barrier of ethene is reduced from 38 to 10 kJ/mol with the absence of two acid sites as active sites), while alkane diffusion is insensitive to acid site density (minor fluctuations of the barrier under different acid sites). The influences of acid site density and the critical HCP species on the diffusion in this work, intertwining with the catalytic mechanism of the MTO reaction, will determine product selectivity.

## CAVITY MODIFICATION FOR SUPER SHAPE SELECTIVITY

### Dynamic multiscale MTO reaction process

Establishing a comprehensive understanding of zeolite shape-selective catalysis in a dynamic and multiscale heterogeneous catalytic process is critically needed in both academic and industry. For zeolite-catalyzed MTO system, even we have presented the principle of cavity-controlled reaction, cavity-controlled coke formation and cavity-controlled diffusion, it is still a very challenging task to explain the whole scene of the MTO process with the time-dependent dynamic behavior with interactive effects of reaction, coke formation and diffusion over 8-MR and cavity-type zeolite materials. This complexity, overlapped with the multiscale reaction and diffusion, makes the proposal of shape-selective strategy an extremely difficult mission.

**Figure 9.**
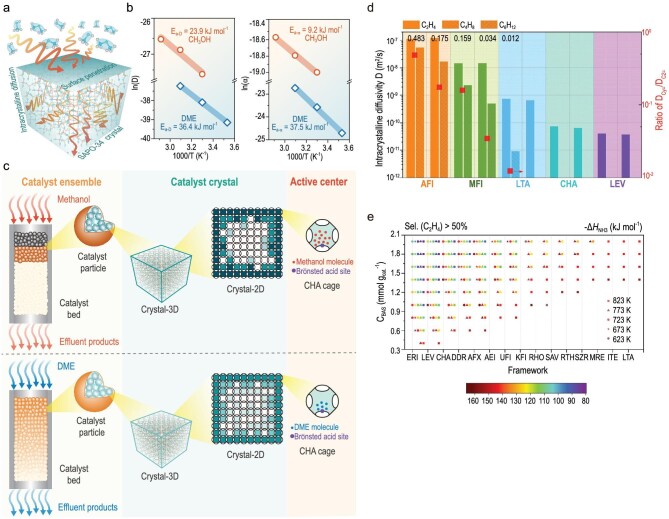
(a) Scheme for diffusion behavior of methanol and Dimethyl ether (DME) over SAPO-34 crystal. (b) Arrhenius plots of intracrystalline diffusivity and surface permeability. (c) Multiscale reaction and deactivation model of MTO and DTO reactions over SAPO-34, from the catalyst-bed scale to the catalyst-crystal and CHA-cavity scale. Adapted from ref. [33]. (d) Intracrystalline diffusivities of binary olefins within AFI, MFI, LTA, CHA and LEV frameworks at 673 K by FFMD simulations. (e) The optimized zeolite framework, BAS density, BAS strength, and operating temperature for the achievement of high selectivity of ethylene (>50%). Adapted from ref. [34] with permission of the American Institute of Chemical Engineers.

Recently, for the methanol and dimethyl ether conversions over SAPO-34, combining diffusion, time-dependent material and reaction, Liu and coworkers [33] disclosed dynamic multiscale cross-talk behaviors. It has been discovered that the cross-talk of catalyst material (coke)-reaction-diffusion (Fig. 9a–c) noticeably regulated the dynamic progress of the dimethylether-to-olefins (DTO) reaction, which differs from the MTO reaction, despite that both reaction are carried out in the same zeolite material and possess very close Hydrocarbon pool mechanisms. Owing to the confined-organics modification, cavity-type SAPO-34 material dynamically evolves during the autocatalytic MTO reaction from initiation to decay, which in turn creates the catalysis with the dynamical evolution of diffusion and reaction. This dynamic cross-talk occurs from the catalyst-bed scale, catalyst crystal and CHA-cavity scale, and ultimately leads to the spatiotemporal heterogeneity in the distribution of carbonaceous compounds at multiple scales, opening up the origin of heterogeneous catalytic efficiency, shape-selective catalysis and the manner of deactivation. Remarkably, in the dynamic reaction course of MTO and DTO, the shape selectivity of 8-MR and cavity-type zeolite materials should be responsible for these multiscale cross-talk behaviors and mechanisms.

The evolution of the zeolite-catalyzed MTO reaction always takes place in a dynamic manner, and the reaction always evolves in a mutual manner, in which the formation of olefin, the accessibility of acid center, the generation of coke species, and the change in mass transfer properties of the catalyst are all acting together to form a dynamic MTO process. During the dynamic evolution of the zeolite-catalyzed MTO reaction, comprehensive spatiotemporal integration of cavity-type catalyst material, diffusion and reaction will effectively promote catalyst optimization and process development.

Furthermore, recently, Liu and coworkers [34] presented a quantitative shape selectivity for zeolite catalysis, which involves thermodynamics, reaction kinetics and molecular diffusion in a confined zeolite framework. The product’s distribution derives from the competitive effect of molecular diffusion and secondary reactions between hydrocarbon compounds, the ratio of selectivity of C_y_^=^/C_2_^=^ and alkane/alkene. Using a combination of MD simulations and projected models, the competitive diffusion between olefin species within zeolite frameworks embellished with various heteroatoms has been thoroughly investigated. With the aid of force field molecular dynamics (FFMD), Fig. 9d–e provided the intracrystalline diffusivities of ethylene, n-butene and n-hexene in binary components of C_2_H_4_/C_4_H_8_ and C_2_H_4_/C_6_H_12_ loaded in AFI, MFI, LTA, CHA and LEV framework at 673 K. For the same 8-MR zeolites with minor difference in maximum free sphere diameter (MFSD), such as LTA with 0.415 nm, CHA with 0.366 nm, and LEV with 0.347 nm, the impact of MFSD on molecular diffusion causes major variations in the diffusion behavior of olefins. The *ab initio* Molecular Dynamics simulations of the competitive diffusion behavior between binary components of C_2_H_4_/C_3_H_6_, C_2_H_4_/C_4_H_8_, C_2_H_4_/C_5_H_10_, and C_2_H_4_/C_6_H_12_ showed that decreasing MFSD not only facilitates competitive diffusion between olefin species but also selectively retards higher olefins in zeolite frameworks to react with BAS, leading to further secondary reactions. Eventually, based on the effect of multidimensional variables provided by the proposed equation for the quantitative shape-selectivity principle, considering the BAS strength, density and reaction temperature, the probability toward ethene, propene and C_4_^+^ hydrocarbon compounds for zeolite-catalyzed MTH reactions over different catalyst materials were estimated. For instance, as shown in Fig. 9d–e, ERI, LEV, CHA, DDR and AFX with an 8-MR window present high-selectivity of ethene, which may be the potential candidate catalysts for ethene production. Moderate operating temperature, zeolite framework with small pores and low BAS density can together contribute to propene formation, offering the guidance of the utilization of zeolite with narrow pores for excellent MTP catalysts. This modeling can present an approximation of the probability distribution of the hydrocarbons over special zeolite in MTH reaction and search for optimal reaction conditions, zeolite structure and acidic properties, which is of crucial importance in commercial processes and fundamental researches.

### Control strategies for optimized shape selectivity in methanol conversion

Methanol conversion is a dynamic autocatalysis process [35,68]. When methanol is fed and contacts with fresh zeolite catalyst, the reaction undergoes autocatalytic initiation, acceleration and deactivation. In this process, the reaction goes through low selectivity in the initial reaction stage and increased selectivity as organic species are formed in cages in the following reaction stage. While organic species modify cages to help achieve high selectivity, excessive accumulation of coke also leads to deactivation, which needs to be avoided. Therefore, to achieve high reactivity and selectivity, simultaneously, it is necessary to realize the spatial and temporal distribution of the confined organic species from cage-scale to crystal-scale to help shape selectivity and efficient reaction to occur. Based on these understandings, some important regulation strategies are proposed to modify the cavity environment of zeolites [94–96]. A core shell-like structure of SAPO-34 modified by zinc cations improved ethene selectivity, and the diffusion limitation of bulky-size organic species lead to ethene formation via lower methylbenzenes and methylnaphthalenes as HCP species. Pre-coking of fresh catalyst [97] and partial regeneration of the coked catalyst, are potential strategies to improve the shape selectivity in industry applications.

Based on the recognition of cavity-controlled reaction, cavity-controlled coke and cavity-controlled diffusion, for the practical MTO industry, pre-coking of fresh catalyst [97] and partial regeneration [98,99] of the coked catalyst were used to enhance light olefin selectivity during MTO conversion over SAPO-34 by controlling coke confined in CHA cavity. A promising 1-butene ‘pre-coking’ strategy to increasing ethene selectivity and their control mechanism of product selectivity was developed [97]. The pre-positioned coke extending the reaction zone toward the SAPO-34 crystal's near-core, increases the amount of available Brønsted acid sites and lengthens the diffusion trajectory of molecules. The higher olefins tend to transfer to active aromatic compounds, leading to substantial improvement in ethene formation.

Steam cracking, as an effective regeneration strategy, has been applied in both the laboratory-scale reactor and fluidized bed reactor-regenerator pilot plant [98]. Converting coking deposition to naphthalenic-based species over SAPO-34 via steam cracking to enhance ethene selectivity was verified, and 85% light olefin selectivity was achieved in the fluidized bed reactor-regenerator pilot experiments. A more complete decoking process of SAPO-34 in steam has been described in detail, giving the acidic cavity induced coke decomposition route in the cavity-type zeolite catalyst [99]. These modulations of product selectivity from the zeolite catalyst’s modification and reaction pretreatment to realize control of the local environment of the cavity structure, which is significant for the optimization of the catalysts and the MTO process.

## CONCLUSION AND OUTLOOK

Among the remarkable progresses made in zeolite catalysis, MTO process has achieved great success in academic and industry applications, which open a new era for olefin production via non-petrochemical resources. Meanwhile, the development of MTO industrial processes has also inspired fundamental research that provides theoretical guidance for optimizing catalysts and reaction processes, enriching our knowledge of C1 chemistry. Molecular sieve catalysts with a complex microenvironment embedding the super-molecule active center have exhibited demonstrable features and advantages in shape selectivity of MTO reaction. Especially for the cavity-type zeolite with small-pore opening, the host cavity microenvironment acts as the reaction unit to induce the dynamic MTO reaction and shape selectivity.

In the catalytic environment of zeolite catalysts with 8-MR and cavity structure, cavity-controlled HCP species generation, cavity-controlled reaction path selection, cavity-controlled dominant product generation, cavity-controlled confined coke deposition and cavity-controlled diffusion behaviors reveals the special host–guest interaction of zeolite materials and the MTO reaction. This highlights the structure-performance relationship of MTO reaction on the zeolite-catalyzed material with 8-MR and cavity-type structure, providing theoretical guidance for the development and regulation of zeolite catalysts and MTO technologies for the new generation of MTO processes, generally, guiding the catalytic process development of C1 chemistry in zeolite catalysis.

Combined with the multiscale and dynamic properties of reaction and catalytic materials in the MTO reaction, the cross-talk of catalyst material (coke)-reaction-diffusion in cavity-type zeolite-catalyzed MTO reactions were proposed to reveal the real shape-selective catalysis with interactive behaviors and mechanism. The key to developing shape-selective catalysts and achieving an efficient process is to establish the best spatiotemporal coordination in the reaction system through mutual echo, mutual modification and mutual guidance among catalyst materials (modified by coke evolution), reaction, and diffusion. The understanding of all the cavity-controlled behaviors, including cavity-controlled catalytic reaction, coke deposition, and diffusion has been applied in the establishment of control strategy for promoting the development and application of new generation of DMTO technology. Pre-coking strategies for highly shape-selective MTO processes have been confirmed in MTO unit of demonstration scale and will be applied in MTO plants in the future.

Shape-selective catalysis is the primary advantage of molecular sieve catalysis, which can be classified as reactant shape selectivity, transition state shape selectivity and product shape selectivity. Through research of cavity-controlled MTO reactions, it has been discovered that all these shape-selective catalysts are embodied together in the MTO reaction. Although methanol reaction in the cavity environment (∼1 nm) with acidity and ∼4 Å window is an extremely complex catalytic reaction system, MTO reaction catalyzed over SAPO-34 with 8-MR and cavity-type structure brings about high shape selectivity for the production of light olefins. For the MTO reaction with multiple reaction pathways and complex polycycle reaction network, understanding the cavity-controlled shape selectivity can enable the selective production of specific target products by utilizing and modification of 8-MR and cavity-structured microporous zeolite catalysts.

The cavity-controlled reaction performance of MTO has inspired the researcher to develop a variety of strategies and promote a renovation of industrial technology. Modification of the cavity and acidity to adjust the microenvironment of zeolites has been used to enhance olefin selectivity by controlling the microenvironment for methanol conversion. For the practical MTO industry, pre-coking of fresh catalyst and partial regeneration of the coked catalyst were used to enhance olefin selectivity during MTO conversion over cavity-type SAPO-34 catalyst by controlling the confined organic species and their spatial distribution.

The future development of new generation MTO catalysts, based on the understanding of the cavity-controlled principle, is to design the special cavity-type catalysts or modify the acidic cavity of zeolite catalysts for the desired product distribution in methanol conversion. Future technical innovation of the MTO process will require accurate control of reaction, coke formation and diffusion in the confined cavity microenvironment to achieve enhanced catalyst stability and product selectivity in the industrial process.
